# Quantifying uptake and completion of pulmonary rehabilitation programs in people with chronic obstructive pulmonary disease known to tertiary care

**DOI:** 10.1177/14799731231224781

**Published:** 2024-01-05

**Authors:** Sarah Hug, Vinicius Cavalheri, Daniel F Gucciardi, Kylie Hill

**Affiliations:** 1 Curtin School of Allied Health, Faculty of Health Sciences, 1649 Curtin University, Perth, Western Australia, Australia; 2 Department of Physiotherapy, Royal Perth Hospital, Perth, Western Australia, Australia; 3 Allied Health, South Metropolitan Health Service, Perth, Western Australia, Australia; 4 Exercise Medicine Research Institute, Edith Cowan University, Perth, Western Australia, Australia; 5 Department of Physiotherapy, Sir Charles Gairdner Hospital, Perth, Western Australia, Australia

**Keywords:** Pulmonary rehabilitation, uptake, attendance, completion, maintenance, attrition, chronic obstructive pulmonary disease

## Abstract

**Background:**

People with symptomatic chronic obstructive pulmonary disease (COPD) benefit from pulmonary rehabilitation programs (PRPs), but program attrition is common.

**Methods:**

For people with COPD who presented to tertiary care and appeared appropriate for a PRP, we prospectively mapped their PRP journey, explored factors influencing attendance to pre-program assessment and captured program attrition.

**Results:**

Of the 391 participants, 31% (95% CI 27 to 36) were referred to a PRP (*n* = 123; age 68 ± 10years, 62 males [50%], FEV_1_ 45 ± 19%predicted). Of those referred, 94 (76% [69 to 84]) attended a pre-program assessment. Ex-smokers and those who had a healthcare professional (HCP) explain they would be referred were more likely to attend a pre-program assessment (odds ratio [95%CI]; 2.6 [1.1 to 6.1]; and 4.7 [1.9 to 11.7], respectively). Of the 94 who attended, 63 (67% [58 to 77]) commenced; and of those who commenced, 35 (56% [43 to 68]) completed a PRP. All who completed (*n* = 35, 100%) were provided at least one strategy to maintain training-related gains.

**Conclusion:**

Attrition occurs throughout the PRP journey. Interactions with HCPs about PRPs positively influenced attendance. Understanding how HCPs can best contextualise PRPs to encourage referral acceptance and uptake is an important area for further work.

## Introduction

Although the benefits of pulmonary rehabilitation programs (PRPs) for people with symptomatic chronic obstructive pulmonary disease (COPD) are well-documented,^1^ these programs are underutilised globally.^2^ A retrospective audit on 7413 people referred to 210 PRPs across England and Wales demonstrated that 69% of people with COPD referred to a PRP attended their pre-program assessment. Of these, 85% commenced and 71% completed the program.^3^ There is a lack of prospective data quantifying the entire journey through a PRP, from referral, attendance to pre-program assessment, commencement, and completion of these programs.

In this study, we prospectively mapped the PRP journey of people with COPD in Perth, Australia. We sought to understand the proportion of people referred to a PRP from tertiary care who attended a pre-program assessment, commenced, and completed a PRP. We also explored factors which influenced attendance to pre-program assessment. Prospectively quantifying uptake and completion of PRPs can assist in the development of strategies to mitigate the implementation gap, so that more people can obtain the benefits associated with PRPs.

## Methods

People with COPD were sequentially recruited from the Respiratory in-patient and out-patient areas of three tertiary hospitals. Ethics approval was obtained at all sites. Those who appeared broadly appropriate for an outpatient group-based PRP (i.e. independently ambulant, be able to understand English, have a life expectancy of greater than 6 months, not residing in supported residential care, and did not have evidence of a cognitive impairment) were included (Supplement 1). For every participant who was referred to a PRP during the 5-months recruitment period, we recorded the number who completed the following milestones: (i) attended a pre-program assessment, (ii) commenced a PRP, (iii) completed a PRP, and (iv) received a strategy to maintain training-related gains. Data were extracted on age, gender, body mass index (BMI), forced expiratory volume in 1 second (FEV_1_), smoking status (current/ex-smoker), living alone (yes/no), recruitment setting (in-patient/out-patient), whether the participant recalled a healthcare professional (HCP) explain they would be referred to a PRP, rating of the enthusiasm with which the referrer spoke about the program (0 to 100 Visual Analogue Scale [VAS])*,* who contacted the participant regarding the referral, location of assessment and reasons why participants did not complete each milestone. Variables were compared between participants who were referred and attended the pre-program assessment versus those who were referred but did not attend the pre-program assessment using independent t-tests or chi-square ( χ ^2^) tests. Variables which differed between groups were included in a logistic regression model. Statistical analyses were performed in Stata 17 (2021 StataCorp LLC, USA).

## Results

**Figure 1.**
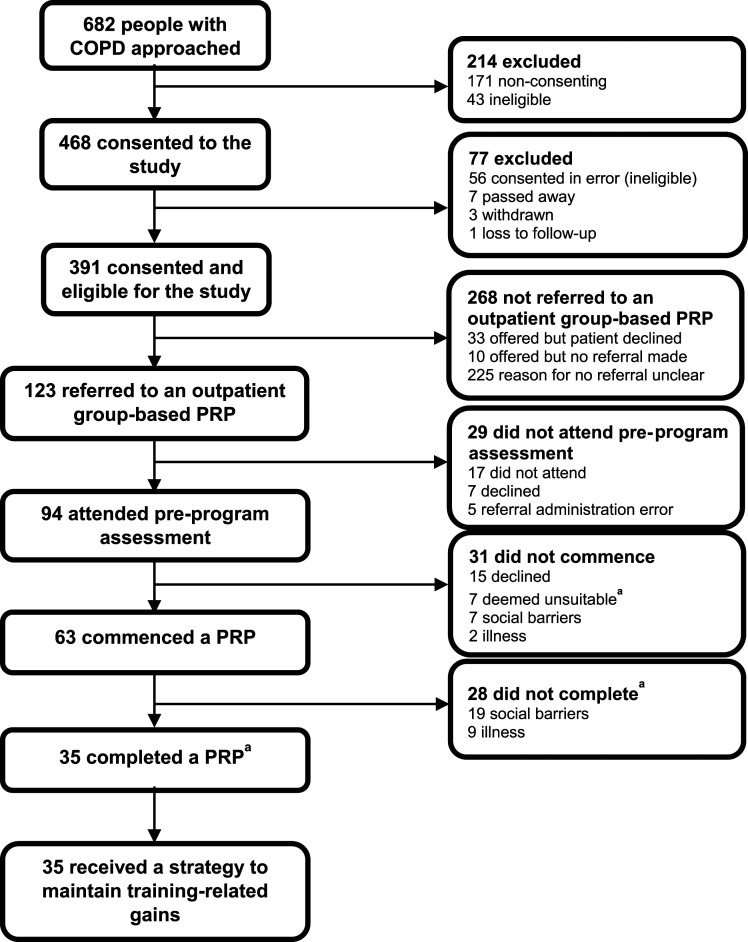
Flow of participants through the study (recruitment over a 5-month period) ^a^as determined by the treating physiotherapist.

Figure 1 presents the flow of participants. Of the 391 participants who consented and were eligible, 123 (31%; 95% confidence interval [CI], 27 to 36) were referred to a PRP (mean ± SD or frequency [%]: age 68 ± 10 years, 62 males [50%], BMI 27±8 kg/m^2^, FEV_1_ 45 ± 19% predicted).

Of the 123 participants referred, 94 (76% [69 to 84]) attended their pre-program assessment. The between group analyses are presented in Table 1. The odds of attending a pre-program assessment were greater in ex-smokers (compared with smokers; odds ratio [95% CI], 2.6 [1.1 to 6.1]) and in those who had a HCP explain that they would be referred (compared with those who did not; 4.7 [1.9 to 11.7]). Of the 94 participants who attended a pre-program assessment, 63 (67% [58 to 77]) commenced a PRP, with 46 (73% [62 to 84]) offered classes twice a week for at least 8 weeks (i.e. a total of 16 sessions). The median [IQR] attendance was 9 [4 to 15] classes. Of the 63 participants who commenced, 35 (56% [43 to 68]) completed a PRP, with all provided at least one maintenance strategy. Common reasons for PRP attrition were participants declining (see Supplement 1), psychosocial barriers (e.g. work/carer commitments, reluctance to attend hospitals or social-phobia) and illness (impact of comorbidities or an exacerbation of their disease).

**Table 1.** Between-group differences in characteristics among those were referred to a PRP and did versus did not attend their pre-program assessment.

Variable	Did not attend (*n* = 29)		Attended (*n* = 94)		MD (95% CI) or χ ^2^, *p* value
Age, yr	67 ± 12		68 ± 9		MD -1.0 (−5.1 to 3.1)
Gender, male (%)	13 (45%)		49 (52%)		χ ^2^ 0.5, *p* = .49
BMI, kg/m^2^	29 ± 11		27 ± 7		MD 1.7 (−1.7 to 5.2)
FEV_1_, % predicted^#^	45 ± 21		45 ± 19		MD 0.5 (−8.4 to 9.5)
Ex-smoker, Y	14 (50%)		67 (72%)		χ ^**2**^ **4.73, *p* = .030**
Living alone, Y	11 (38%)		32 (34%)		χ ^2^ 0.15, *p* = .70
Recruited as an out-patient, Y	13 (45%)		54 (57%)		χ ^2^ 1.4, *p* = .23
HCP explained referral to PRP, Y	11 (38%)		72 (77%)		χ ^**2**^ **15.1, *p* < .001**
	Consultant	4 (14%)	Consultant	25 (27%)	
	Registrar	10 (35%)	Registrar	24 (26%)	
HCP initiating referral	Resident	0	Resident	2 (2%)	χ ^2^ 3.79, *p* = .58
	PT	14 (48%)	PT	38 (40%)	
	Other	1 (3%)	Other	5 (5%)	
Referrer enthusiasm (VAS 0 to 100)^a^	70 ± 22		76 ± 22		MD -6 (−21 to 9)
	PT	12 (41%)	PT	45 (48%)	
Who contacted the participant regarding the referral?^#^	Admin	12 (41%)	Admin	46 (49%)	χ ^2^ 1.27, *p* = .74
	Unsure	5 (18%)	Unsure	3 (3%)	
Site change, Y	9 (31%)		38 (40%)		χ ^2^ 0.83, *p* = .36

Footnotes: Data expressed as mean ± SD, median [IQR] or *n* (%). Boldface indicates a significant difference between groups.

Abbreviations: Admin – administration staff, Ax – assessment, CI – confidence interval, HCP – healthcare professional, MD – mean difference, OR – unadjusted odds ratio, Site change – whether the pre-program assessment took place at a different site from where the referral was initiated, PRP – pulmonary rehabilitation program, PT- Physiotherapist, VAS – Visual Analogue Scale, Y – yes, χ ^2^ – chi squared test statistic.

^a^indicates missing observations.

## Discussion

Non-referral remains the primary hurdle to PRP utilisation,^3,4^ but attrition was problematic throughout the rehabilitation journey. Approximately 24% of those referred did not attend a pre-program assessment, despite majority of these ‘non-attenders’ appearing *suitable* for the program.^4^ Although our data revealed ex-smokers were 2.6 times more likely to attend the pre-program assessment, 50% of ex-smokers were also non-attenders. Participants who had the referral explained by a HCP were 4.7 times more likely to attend their pre-program assessment. That is, the odds of the referral being accepted by the person was substantially greater when, at the point of referral (i.e. during the acute admission or during the out-patient Respiratory Medicine appointment where shared-decision making occurs), there was an interaction with a HCP about the referral to a PRP. This novel finding reinforces earlier recommendations that PRPs should take precedence when discussing treatment options with people with COPD.^3^ Healthcare professionals should help people with COPD to contextualise the evidence underpinning PRPs to individual circumstance to promote referral acceptance. Despite the known benefits of PRPs,^1,2^ we found people continue to decline these programs. Our earlier work demonstrated that among people with COPD, low interest in participating in a PRP may stem from underlying feelings of unworthiness.^4^ Therefore, in people who appear ‘unmotivated or disinterested’ regarding a PRP, the HCP should consider carefully probing feelings of shame that may lead them to decline healthcare.

Once assessed, 33% did not follow through and commence a PRP. Reasons for attrition included participants declining, illness or psychosocial barriers. Similarly, illness and psychosocial barriers were common reasons for attrition among non-completers (44%). Some of these barriers may be overcome by HCPs offering people with COPD a choice of alternative methods of PRP delivery.^2,3^

Strengths of this study include sequential recruitment and prospective follow-up of participants which provided insight to the reasons for attrition. We would have liked to explore factors influencing commencement and completion of PRPs, but attrition limited our capacity to do so. Further, we were only able to account for participants referred to a PRP from the three study hospitals, which is a limitation of the recruitment strategy. Although recruitment to this study occurred during the coronavirus 2019 (COVID-19) pandemic, community transmission during this time in Perth, Western Australia was negligible. Of note, COVID-19 was not reported as the reason for drop out by participants or physiotherapists who delivered the programs. Our decision to exclude non-English speaking participants is a limitation as these people are likely to face additional barriers to program uptake and completion. A detailed qualitative analysis would help to understand the determinants of behaviours underpinning the uptake (or otherwise) of each milestone explored in this study.

Attrition from PRPs occurs throughout the rehabilitation journey. Healthcare professional interactions about PRPs can have an influence on referral acceptance. Understanding how HCPs can best contextualise PRPs to encourage referral acceptance and uptake is an important area for further work. People who decline a PRP may be masking a belief that they are unworthy of healthcare. Targeting this unhelpful belief may represent a pathway to optimise uptake.

## Supplemental Material


Supplemental Material - Quantifying uptake and completion of pulmonary rehabilitation programs in people with chronic obstructive pulmonary disease known to tertiary care
Supplemental Material for Quantifying uptake and completion of pulmonary rehabilitation programs in people with chronic obstructive pulmonary disease known to tertiary care by Sarah Hug, Vinicius Cavalheri, Daniel F Gucciardi and Kylie Hill in Chronic Respiratory Disease.

## Data Availability

The data that support the findings of this study are available from the corresponding author upon reasonable request and approval from the Human Research Ethics Committee.
