# Supplementary material for: Quantifying uptake and completion of pulmonary rehabilitation programs in people with chronic obstructive pulmonary disease known to tertiary care
Source: Chron Respir Dis. 2024 Jan 5;21:14799731231224781. doi: 10.1177/14799731231224781 (PMC10771047; doi:10.1177/14799731231224781)
Supplement: Supplemental Material - Quantifying uptake and completion of pulmonary rehabilitation programs in people with chronic obstructive pulmonary disease known to tertiary care [file sj-pdf-1-crd-10.1177_14799731231224781.pdf]

Title: Quantifying uptake and completion of pulmonary rehabilitation programs in people with COPD known to tertiary care.

Authors:

Sarah Hug

Curtin School of Allied Health, Faculty of Health Sciences, Curtin University, Perth, Western Australia, Australia.

Department of Physiotherapy, Royal Perth Hospital, Perth, Western Australia, Australia.

sarah.dcosta@postgrad.curtin.edu.au

A/Professor Vinicius Cavalheri

Curtin School of Allied Health, Faculty of Health Sciences, Curtin University, Perth, Western Australia, Australia.

Allied Health, South Metropolitan Health Service, Perth, Western Australia, Australia.

Exercise Medicine Research Institute, Edith Cowan University, Perth, Western Australia, Australia.

vinicius.cavalheri@curtin.edu.au

Professor Daniel F. Gucciardi

Curtin School of Allied Health, Faculty of Health Sciences, Curtin University, Perth, Western Australia, Australia.

d.gucciardi@curtin.edu.au

Professor Kylie Hill [corresponding author]

Curtin School of Allied Health, Faculty of Health Sciences, Curtin University, Perth, Western Australia, Australia.

Department of Physiotherapy, Sir Charles Gairdner Hospital, Perth, Western Australia, Australia.

Address: Curtin School of Allied Health, Curtin University, GPO Box U1987, Perth, WA 6845, Australia.

Phone: +61 8 9266 2774

Email: k.hill@curtin.edu.au

Supplementary material

Data reported in this prospective observational study (i.e. horizon scan) were collected as part of a larger mixed-methods study.[1] The purpose of the larger study is to optimise the implementation and utilisation of pulmonary rehabilitation programs (PRPs) for people with COPD known to tertiary care in Perth, Western Australia (WA). Further information for this study can be found at our project page on the Open Science Framework (OSF); https://osf.io/qet25/.

Methods

Inclusion criteria

People with COPD were sequentially recruited from the Respiratory in-patient and out-patient areas of three tertiary hospitals in Perth, WA between August 2020 and January 2021. The inclusion and exclusion criteria for this horizon scan study were designed to find people likely to be appropriate for referral to a physiotherapist-led PRP conducted in either a hospital out-patient department or community centre. That is, participants were required to have a diagnosis of COPD, be English speaking, be independently ambulant and have a life expectancy of more than six months. Potential participants were excluded if they were living in supported residential aged care or had a cognitive impairment. Those recruited as out-patients were excluded if they had participated in either a PRP within the previous 12 months, or a 'maintenance' PRP within the previous 8 weeks. Of those who consented to the larger study, those referred to a face-to-face group-based PRP during the five-month recruitment period were included in this study.

Grouping of participants

Data pertaining to referral behaviours has been published separately.[2] In this previous study which reported referral behaviours,[2] the research team utilised pragmatic criteria to group participants according to their suitability for a referral to a PRP. These criteria were based on the participant having symptoms limiting their physical activity and no comorbidities precluding safety during exercise, which is appropriate for PRP service delivery in Perth, WA. The research aim for the previous study was to understand what proportion of people with COPD who are receiving specialist care through a tertiary hospital and appear suitable for referral to a centre-based PRP went on to be referred within two weeks of hospital discharge or out-patient appointment.[2] Grouping according to suitability for a referral to a PRP enabled us to understand which participant referrals had been missed (which is the implementation gap) and which participants were appropriately not referred. In the current analyses, the research aim was, among those who were referred to a PRP (regardless of their suitability of the program), to quantify the proportion who went on to attend a pre-program assessment, commence and complete a PRP. We therefore did not apply the suitability grading. Further, any participant who was referred to a PRP at any time during the five-month recruitment period was included in study analyses.

Data collection

Recruitment and data collection were completed by research officers, all of whom were qualified Physiotherapists, with at least 10 years of clinical experience (either within Respiratory Medicine or specific to a PRP). Research officers received

standardised training for interviewing participants and were given strict instruction to observe and interact with participants only. Data for initial recruitment were collected from the medical records and via an interview with the participant using REDCap data collection tools (available at our OSF webpage). Once recruited, data regarding the journey from point of referral, through to attendance at pre-program assessment, program commencement and program completion (including the provision of strategies for program maintenance) were prospectively tracked by the first author via discussion with the PRP physiotherapist and/or participant and reviewing the medical records. If a participant did not attend, commence or complete a PRP, reasons for attrition were sought from the participant and/or treating physiotherapist, and recorded in our REDCap data collection tools. The aim of this study was to observe and quantify current clinical practice of PRPs across WA. Therefore PRP prescription and program completion was determined by the treating physiotherapist, the study reported on these data.

Exploring the interaction with a healthcare professional

During the initial recruitment interview, the research officer asked the participant the following question:

1. Did a healthcare professional explain to you that you will be referred to a pulmonary rehabilitation program (exercise classes for people with lung problems)?

If a participant was not offered a referral to a PRP or did not recall a healthcare professional offering them a referral to a PRP, this question was marked as ‘no’ or

‘unable to determine’ respectively. If the participant recalled being referred to a PRP, they were asked to complete the following:

2. Please rate the level of enthusiasm with which the healthcare profession (who spoke with you about the referral) talk about the rehabilitation program (where 0 means no enthusiasm and 100 means the highest possible enthusiasm) (graded using a 0 to 100 Visual Analogue Scale [VAS][3]).

These requests were read by the research officer verbatim and were asked after the interaction with the healthcare professional.

Data analysis

The variables which were included in the analyses for this study were purposefully selected by the research team through clinical judgement. These variables were easily measured by clinicians, and if found to differ between groups, could be used to inform clinical practice and potentially improve engagement with PRPs.

As this study was exploratory (rather than hypothesis-testing) no formal sample size calculations were undertaken. It is important to note that we would have liked to undertake similar analyses to explore factors influencing whether or not a participant commenced, and completed a PRP, however significant attrition limited our capacity to do so.

Overview of reasons participants declined to attend a pre-program assessment

- Overwhelmed by multiple recent hospital admissions
- Already doing own exercises and feels active enough
- Not wanting to attend various different venues for appointments
- No energy and problems with weight loss

Overview of reasons participants declined to commence a PRP

- Already done PRP in past
- Too much walking involved
- Carer duties for family members
- Preference to complete a home exercise program instead
- Dislike of exercising in group settings
- Competing medical issues i.e. upcoming radiotherapy for lung cancer
- Dislike of exercising in the hospital setting which was a place of employment for one participant (felt uncomfortable seeing ex-colleagues)
- Past negative experiences with PRP Physiotherapy staff
- Wanting to exercise at own pace rather than be pushed in the class setting
- Feeling overwhelmed from mental health issues – seems too much to deal with.
- “I don’t want to be in a hospital unless I have too i.e. I am dying.”
- It’s too far to drive there twice a week
- Previously attended a PRP and contracted two chest infections
- Not interested in attending as too many appointments to keep up with

References

1. Hug S, Cavalheri V, Gucciardi DF, et al. OPTImising the implementation of pulMonary rehAbiLitation in people with chronic obstructive pulmonary disease (the OPTIMAL study): mixed methods study protocol. *BMC Pulmonary Medicine* 2020; 20. DOI: 10.1186/s12890-020-01322-4.
2. Hug S, Cavalheri V, Gucciardi DF, et al. An evaluation of factors that influence referral to pulmonary rehabilitation programs among people with COPD. *Chest* 2022. DOI: https://doi.org/10.1016/j.chest.2022.01.006.
3. Wewers ME and Lowe NK. A critical review of visual analogue scales in the measurement of clinical phenomena. *Research in Nursing & Health* 1990; 13: 227-236. DOI: 10.1002/nur.4770130405.
4. von Elm E, Altman DG, Egger M, et al. The Strengthening the Reporting of Observational Studies in Epidemiology (STROBE) statement: guidelines for reporting observational studies. *J Clin Epidemiol* 2008; 61: 344-349. 2008/03/04. DOI: 10.1016/j.jclinepi.2007.11.008.

e-Appendix 1. STROBE checklist[4]

STROBE Statement—checklist of items that should be included in reports of observational studies

	Item No	Recommendation	Page No
**Title and abstract**	1	(*a*) Indicate the study's design with a commonly used term in the title or the abstract	2
		(*b*) Provide in the abstract an informative and balanced summary of what was done and what was found	2
**Introduction**			
Background/rationale	2	Explain the scientific background and rationale for the investigation being reported	3
Objectives	3	State specific objectives, including any prespecified hypotheses	3
**Methods**			
Study design	4	Present key elements of study design early in the paper	3,4
Setting	5	Describe the setting, locations, and relevant dates, including periods of recruitment, exposure, follow-up, and data collection	3,4 and Suppl.1
Participants	6	(*a*) *Cohort study*—Give the eligibility criteria, and the sources and methods of selection of	4 and Suppl.1

		participants. Describe methods of follow-up<br>*Case-control study*—Give the eligibility criteria, and the sources and methods of case ascertainment and control selection. Give the rationale for the choice of cases and controls<br>*Cross-sectional study*—Give the eligibility criteria, and the sources and methods of selection of participants	
		(*b*) *Cohort study*—For matched studies, give matching criteria and number of exposed and unexposed<br>*Case-control study*—For matched studies, give matching criteria and the number of controls per case	N/A
Variables	7	Clearly define all outcomes, exposures, predictors, potential confounders, and effect modifiers. Give diagnostic criteria, if applicable	4 and Suppl.1
Data sources/ measurement	8*	For each variable of interest, give sources of data and details of methods of assessment (measurement). Describe comparability of assessment methods if there is more than one group	4 and Suppl.1
Bias	9	Describe any efforts to address potential	Suppl.1

		sources of bias	
Study size	10	Explain how the study size was arrived at	Suppl.1
Quantitative variables	11	Explain how quantitative variables were handled in the analyses. If applicable, describe which groupings were chosen and why	4
Statistical methods	12	(*a*) Describe all statistical methods, including those used to control for confounding	4 and Suppl.1
		(*b*) Describe any methods used to examine subgroups and interactions	N/A
		(*c*) Explain how missing data were addressed	N/A
		(*d*) *Cohort study*—If applicable, explain how loss to follow-up was addressed<br>*Case-control study*—If applicable, explain how matching of cases and controls was addressed<br>*Cross-sectional study*—If applicable, describe analytical methods taking account of sampling strategy	N/A
		(*e*) Describe any sensitivity analyses	N/A
**Results**			
Participants	13*	(a) Report numbers of individuals at each stage of study—e.g. numbers potentially eligible, examined for eligibility, confirmed	4,5<br>Fig. 1

		eligible, included in the study, completing follow-up, and analysed	
		(b) Give reasons for non-participation at each stage	Fig. 1
		(c) Consider use of a flow diagram	Fig. 1
Descriptive data	14*	(a) Give characteristics of study participants (e.g. demographic, clinical, social) and information on exposures and potential confounders	4, 5<br>Table 1
		(b) Indicate number of participants with missing data for each variable of interest	Table 1
		(c) *Cohort study*—Summarise follow-up time (e.g., average and total amount)	4 and Suppl.1
Outcome data	15*	*Cohort study*—Report numbers of outcome events or summary measures over time	5<br>Fig. 1<br>Table 1
		Case-control study—Report numbers in each exposure category, or summary measures of exposure	N/A
		Cross-sectional study—Report numbers of outcome events or summary measures	N/A
Main results	16	(*a*) Give unadjusted estimates and, if	4,5

		applicable, confounder-adjusted estimates and their precision (e.g., 95% confidence interval). Make clear which confounders were adjusted for and why they were included	Table 1
		(*b*) Report category boundaries when continuous variables were categorized	N/A
		(*c*) If relevant, consider translating estimates of relative risk into absolute risk for a meaningful time period	N/A
Other analyses	17	Report other analyses done—e.g. analyses of subgroups and interactions, and sensitivity analyses	N/A
**Discussion**			
Key results	18	Summarise key results with reference to study objectives	6
Limitations	19	Discuss limitations of the study, considering sources of potential bias or imprecision. Discuss both direction and magnitude of any potential bias	7
Interpretation	20	Give a cautious overall interpretation of results considering objectives, limitations, multiplicity of analyses, results from similar	7

		studies, and other relevant evidence	
Generalisability	21	Discuss the generalisability (external validity) of the study results	7
**Other information**			
Funding	22	Give the source of funding and the role of the funders for the present study and, if applicable, for the original study on which the present article is based	1

N/A = Not applicable
